# A Machine Learning Approach for Detecting Vicarious Trial and Error Behaviors

**DOI:** 10.3389/fnins.2021.676779

**Published:** 2021-07-07

**Authors:** Jesse T. Miles, Kevan S. Kidder, Ziheng Wang, Yiru Zhu, David H. Gire, Sheri J. Y. Mizumori

**Affiliations:** ^1^Graduate Program in Neuroscience, University of Washington, Seattle, WA, United States; ^2^Psychology Department, University of Washington, Seattle, WA, United States; ^3^Undergraduate Neuroscience Program, University of Washington, Seattle, WA, United States

**Keywords:** hippocampus, vicarious trial and error, VTE, machine learning, decision-making, neural oscillations, theta, gamma

## Abstract

Vicarious trial and error behaviors (VTEs) indicate periods of indecision during decision-making, and have been proposed as a behavioral marker of deliberation. In order to understand the neural underpinnings of these putative bridges between behavior and neural dynamics, researchers need the ability to readily distinguish VTEs from non-VTEs. Here we utilize a small set of trajectory-based features and standard machine learning classifiers to identify VTEs from non-VTEs for rats performing a spatial delayed alternation task (SDA) on an elevated plus maze. We also show that previously reported features of the hippocampal field potential oscillation can be used in the same types of classifiers to separate VTEs from non-VTEs with above chance performance. However, we caution that the modest classifier success using hippocampal population dynamics does not identify many trials where VTEs occur, and show that combining oscillation-based features with trajectory-based features does not improve classifier performance compared to trajectory-based features alone. Overall, we propose a standard set of features useful for trajectory-based VTE classification in binary decision tasks, and support previous suggestions that VTEs are supported by a network including, but likely extending beyond, the hippocampus.

## 1. Introduction

Introduced and popularized in the 1930s, vicarious trial and error (VTE) is a well documented behavioral phenomenon where subjects vacillate between reward options before settling on their final choice (Muenzinger and Gentry, 1931; Tolman, 1938). This behavior is best understood in rats making decisions to go left or right, and as such, VTE trajectories tend to have curves that change direction at decision points. Current theories claim that subjects mentally assess possible options before making a final decision during VTEs (Redish, 2016), suggesting that they may be related to, but not necessarily identical to, an underlying deliberative process. While such a relationship to deliberation is complex and outside of the scope of this paper, it is clear that VTEs are a valuable behavioral variable to take into account when studying decision-making, particularly when investigating neural processing during decisions.

The majority of recent experiments examining the neural underpinnings of VTEs have focused on the rodent hippocampus (HPC). Bilateral electrolytic HPC lesions decrease the mean number of VTEs in a visual discrimination task, particularly during early learning (Hu and Amsel, 1995); though see Bett et al. (2012). Similarly, bilateral ibotenic acid HPC lesions decrease the number of VTEs rats exhibit before they have located a reward in a spatial task (Bett et al., 2012). In addition to lesion studies, several electrophysiological findings link the HPC to VTEs. Dorsal HPC recordings during VTEs show serial sweeps of place cell sequences, which first trace the initial direction of the VTE before sweeping in the direction a rat ends up choosing (Johnson and Redish, 2007). Furthermore, dorsal HPC place cell recordings are more likely to represent locations of an unchosen option during VTEs than non-VTEs (Papale et al., 2016). There is also evidence that the field potential oscillation recorded from dorsal HPC differs on decisions where VTEs do and do not occur. In particular, characteristics of HPC theta (4–12 Hz) oscillations, such as its shape and duration, appear to be altered during VTEs, as do aspects of gamma-band (35–100 Hz) oscillations (Amemiya and Redish, 2018; Schmidt et al., 2019), but see Dvorak et al. (2018).

Despite decades-long interest and their utility as a behavioral marker of a putative cognitive process, VTEs have been studied by only a small number of labs. We suspect part of the reason they have not received more attention is that VTE trajectories can be highly variable, which makes it difficult to identify them algorithmically (Goss and Wischner, 1956). The Redish lab has proposed the zIdPhi metric, which quantifies changes in heading angles as rats traverse choice points, for identifying VTEs (Papale et al., 2012, 2016; Amemiya and Redish, 2016, 2018; Redish, 2016; Schmidt et al., 2019; Hasz and Redish, 2020). While successful in their hands, zIdPhi, admittedly, “does not provide a sharp boundary between VTE and not” (Papale et al., 2016, Supplementary Material, section Experimental Procedures). Here we show that standard machine learning models trained on data from a spatial delayed alternation task are able to robustly and reliably distinguish VTE trajectories from non-VTEs in such a task.

Additionally, we assess how the same types of classifier models perform when trained on features of the dorsal HPC oscillation that have been shown to differ between VTEs and non-VTEs (e.g., differences in gamma power and theta wave shape; Amemiya and Redish, 2018; Schmidt et al., 2019). In doing so, we demonstrate that these features are indeed able to separate decision types better than would be expected by random binary classification, though with worse performance than trajectory-based features. Furthermore, we show that providing a classifier with HPC oscillatory dynamics from when animals make choices yields better performance than oscillations from the immediately preceding delay interval, which is when information about the previous choice would need to be held in memory. We also show that a more comprehensive description of the HPC oscillation, the power spectrum, does not perform any better than the model trained on curated features. Finally, we demonstrate that combining informative trajectory- and oscillation- based features does not change classifier performance when compared to classification using trajectory features alone, leading us to conclude that the HPC oscillation does not contain information that complements what can be extracted from the trajectories.

## 2. Methods

### 2.1. Behavioral Task

Food restricted (85% of body weight) Long Evans rats (*n* = 9, Charles River Laboratories) were trained on a previously described spatial delayed alternation task (SDA) task (Baker et al., 2019; Kidder et al., 2021). Briefly, sessions were run on an elevated plus maze (black plexiglass arms, 58 cm long × 5.5 cm wide, elevated 80 cm from floor), with moveable arms and reward feeders controlled by custom LabView 2016 software (National Instruments, Austin, TX, USA). Each trial consisted of a rat leaving its starting location in a randomly chosen “north” or “south” arm, then navigating to an “east” or “west” arm for a 45 mg sucrose pellet reward (TestDiet, Richmond, IN, USA). Sessions typically consisted of 60 trials, though not all trials were used for analysis (see section 2.6 for details). Rewards were delivered when rats alternated from their previously chosen arm (i.e., if they selected the “east” arm on trial *n*−1 then they had to select the “west” arm for reward on trial *n*). After making a choice, rats had the opportunity for reward consumption (if correct) before they returned to a randomly assigned start arm and entered into a 10 s delay period preceding the next trial. Based on this structure, we divided the task into three epochs—choice, return, and delay. The choice epoch was defined as the period when rats left the start arm up until they chose an “east” or “west” reward arm. The return epoch was the period between when the rats were rewarded (or not, depending on their choice) and when they entered the randomly chosen start arm for the upcoming trial. The delay epoch was the 10 s after the animal entered the randomly chosen start arm (before the next choice epoch began). All animal care was conducted according to guidelines established by the National Institutes of Health and approved by the University of Washington's Institute for Animal Care and Use Committee (IACUC).

### 2.2. Microdrive Implantation

Micro-drive bodies were 3-D printed (Form 2 Printer; Formlabs, Sommerville, MA) to contain between 8 and 16 gold plated tetrodes (nichrome; SANDVIK, Sandviken, Sweden), which were implanted unilaterally into the CA1 region of HPC (AP: −3.0, M/L: ±2.0 mm, D/V: −1.8 mm). One animal had two optic fibers implanted bilaterally into the medial prefrontal cortex (mPFC), as well as AAV carrying the Jaws photostimulation construct injected into the mPFC, and was used for additional experiments. The remainder (6) also had tetrodes implanted into the ipsilateral lateral habenula for additional experiments. All animals ran the same behavioral task, and data used for this study were from before any optogenetic stimulation was ever delivered. Tetrodes were connected to a 64-channel Open Ephys electrode interface board (EIB) (open-ephys.org). To eliminate external noise, drive bodies were shelled in plastic tubes lined with aluminum foil coated in a highly conductive nickel spray. One ground wire connected the shell with the EIB and, during surgery, another ground wire was implanted near the cerebellum just inside the skull. After surgery, rats were allowed to recover for ~7 days before entering into testing, and HPC tetrodes were lowered over the course of several days until at least one tetrode showed an oscillation consistent with the CA1 fissure (high-amplitude, asymmetric theta).

### 2.3. Data Acquisition

#### 2.3.1. Behavior Tracking

Two LEDs were attached to either the rat's microdrive or the tethers plugged into the microdrive's headstage before recordings. Rat locations were determined by subtracting a background image taken at the beginning of the session from each frame. Pixels containing the LEDs showed an above threshold difference in brightness, which allowed us to determine rat head locations in each frame. Camera frames were recorded at ~35 Hz using a SONY USB web camera (Sony Corporation, Minato, Tokyo). Frames were time-stamped with a millisecond timer run by LabView and sent to the Open Ephys acquisition software (open-ephys.org) for later alignment of electrophysiological and position information.

#### 2.3.2. Electrophysiology

Electrophysiological data were sampled at 30 kHz using Intan headstages (RHD2132; Intan Technologies, Los Angeles, California) connected to the Open-Ephys EIB. Digitized signals were sent via daisy chained SPI cables through a motorized commutator that prevented tether twisting (AlphaComm-I; Alpha Omega Co., Alpharetta, GA) and into an Open-Ephys acquisition board (open-ephys.org). All further processing and filtering was done offline using custom MATLAB scripts (see section 2.4.2 for more details).

### 2.4. Classifier Features

#### 2.4.1. Trajectory-Based Features

Trajectories on our maze always started from either a “north” or “south” arm, ended on either an “east” or “west” arm, and required rats to traverse narrow bridges between arms and a center platform (see section 2.1 for more details). Furthermore, we attached the LEDs used for tracking to the microdrives implanted on the rat's head, and record from above the maze. As such, our features were optimized for behaviors where heads are being tracked from above along narrow corridors that require a large change in the orientation of motion to get from a starting point to ending point. For example, we suspect the same features can be used to identify VTE behaviors on binary decision tasks run on T-mazes, Y-mazes, and other mazes where searchers must change their orientation of motion toward the left or right as they make their decision. It is yet to be determined whether the features we describe for this paper would be suitable for tasks like radial arm mazes or Barnes mazes, where searchers usually exhibit complex trajectories due to the multitude of choice options.

We calculated seven features of choice epoch trajectories—the standard deviation (SD) of the x-position (*x*_σ_), the SD of the y-position (*y*_σ_), the trial's z-scored, integrated change in heading angle (*zIdPhi*), the trial duration (*dur*), how well the trial was fit by a sixth degree polynomial (*r*^2^), and the number of Fourier coefficients needed to describe the fit of the polynomial (*n*_*coef*_). Both *x*_σ_ and *y*_σ_ were calculated using the std method from Python's numpy package for the x and y position vectors of the rat's trajectory on a given trial. The *IdPhi* score for a trial was defined as:

(1) $$\begin{array}{l} \phi =arctan2\left(dy,dx\right) \end{array}$$

(2) $$\begin{array}{l} IdPhi=\overset{b}{\underset{i=a}{\sum }}|\phi_{a}-\phi_{a-1}| \end{array}$$

where *arctan*2 is the 2-argument arctangent function and *dx* and *dy* are changes in the trajectory's x and y position, respectively. This value was transformed to *zIdPhi* by converting to a z-score, which was calculated for each session individually. We set the *zIdPhi* threshold value, above which something was assigned as a VTE, by iterating through values from the 50th to the 80th percentile and choosing the value that maximized area under the receiver operating characteristic (ROC) curve. The *dur* feature measures the duration a rat was within an experimenter defined choice point on the maze. The *r*^2^ value was determined using a two-step process. First, optimal coefficients for each of the terms in the polynomial were calculated using the curve_fit method of the scipy.optimize package with the vector of x positions as the independent variable and the vector of y positions as the dependent variable. From here, we used the optimized outputs as inputs to a generic sixth degree polynomial function, calculated the error sum of squares between the observed y values and modeled outputs (*SSE*, see Equation 3), and calculated the total sum of squares (SST, see Equation 4). The calculation of the *r*^2^ value is shown in Equation (5).

(3) $$\begin{array}{l} SSE=\overset{n}{\underset{i=1}{\sum }}{\left(y_{i}-\hat{y_{i}}\right)}^{2} \end{array}$$

(4) $$\begin{array}{l} SST=\overset{n}{\underset{i=1}{\sum }}{\left(y_{i}-ȳ\right)}^{2} \end{array}$$

(5) $$\begin{array}{l} r^{2}=1-\frac{SSE}{SST} \end{array}$$

In (3), $\hat{y_{i}}$ is the estimated y position at the *i*-th location in the trajectory, and ȳ is the mean y position of the trajectory in (4). We noticed that plotting the polynomial estimates with poor fits (which were mainly VTEs) created a trajectory that looked similar to a damped oscillation, so we devised the *n*_*coef*_ feature—which is the number of Fourier coefficients needed to describe the polynomial fit estimate—to capture this oscillatory character. Intuitively, higher values of *n*_*coef*_ were expected to correlate with instances of VTE.

#### 2.4.2. Oscillation-Based Features

To quantify features of the HPC CA1 oscillation, we down-sampled our data by a factor of 30, going from a 30 kHz sampling rate to a 1kHz sampling rate, and z-scored the downsampled timeseries to put amplitude in units of standard deviations. Based on previous work (Amemiya and Redish, 2018; Schmidt et al., 2019), we were interested to see if we could use features of the HPC CA1 oscillation to classify VTE vs non-VTE trials. We used seven features—the asymmetry index (*AI*) of the wide-band theta oscillation, average ascending (*asc*) and descending (*desc*) durations of the wide-band theta oscillation, the average (normalized) low and high gamma powers (*LG* and *HG*, respectively), the cycle-averaged gamma ratio (*GR*), and the average duration of a theta cycle. Each trial had multiple measurements of each value, so we also used the SD of these measurements as a feature for all but the *asc* and *desc* features, giving a 12-variable feature vector for each trial.

Previous reports have demonstrated asymmetric theta oscillations in different layers of the HPC (Buzsáki et al., 1985, 1986; Buzsáki, 2002), so we used a low-pass filtered signal with the cutoff frequency at 80 Hz to identify peaks and troughs of the theta oscillation as well as the ascending duration, descending duration, and total duration of each theta cycle (Belluscio et al., 2012; see **Figure 3A** for an example). We define a theta cycle as beginning at identified peaks in the low-pass filtered signal, and require peaks be separated by at least 0.0833 s (the upper frequency range of the theta band). The *AI* is defined as:

(6) $$\begin{array}{l} AI=log\left(asc\right)-log\left(desc\right) \end{array}$$

such that cycles with longer ascending than descending durations will give positive values, cycles with equal ascending and descending durations will equal 0, and cycles with shorter ascending than descending durations will give negative values. Because different HPC recording locations can have differently shaped theta oscillations (Buzsáki et al., 1985, 1986; Buzsáki, 2002), we ensured that all days used for analysis had *AI* distributions that were skewed in the same direction.

To estimate gamma powers, first we bandpass filtered our downsampled timeseries between 35 and 55 Hz for low gamma and 61–100 Hz for high gamma using third order, zero-lag Butterworth filters. These values were then z-scored, putting units of amplitude into standard deviations. The power in a gamma-band timeseries, g(t), was estimated using:

(7) $$\begin{array}{l} p\left(t\right)=|\tilde{g}\left(t\right){|}^{2} \end{array}$$

where $\tilde{g}\left(t\right)$ denotes the Hilbert transform of *g*(*t*). We then used these power estimates to calculate cycle-by-cycle *GRs*. For a given cycle, the gamma ratio was defined as:

(8) $$\begin{array}{l} GR=\frac{\hat{LG}\left(t\right)}{\hat{HG}\left(t\right)} \end{array}$$

where the hat denotes the time average of the bandpassed gamma power trace across a theta cycle. The gamma ratio for the entire trial was the average of these cycle-by-cycle values.

Oscillation-based features were calculated for both delay epochs and choice epochs, and classifiers were trained and tested on data from both of these epochs separately.

#### 2.4.3. Power Spectral Density

In addition to pre-defined oscillation bands and bandpass filtering signals, we performed the same classifier-based analysis of neural data using power spectral density (PSD) estimates as features instead of the curated oscillation features. For this, we used MATLAB's periodogram function (version 2018 B; MathWorks, Nattick, MA), with a Hamming window over the duration of the signal, a frequency resolution of 1 Hz, and a range of 1–100 Hz. To maintain consistency with curated oscillation features, we use the z-transformed HPC oscillation. PSD estimates are kept as original values, as opposed to the common decibel conversion. Classifiers were trained on PSD estimates obtained from choice epoch data.

### 2.5. Classifier Implementation

#### 2.5.1. Classifier Models

We used the scikit_learn library from Python to create and test k-nearest neighbor (KNN) and support vector machine (SVM) models. All instances of the KNN model used 5 neighbors for classification, though results for 3–10 neighbors did not lead to different conclusions. All instances of the SVM model used a radial basis function (RBF) kernel for assessing distance/similarity. A γ parameter dictates the width and shape of the RBF, with lower values giving wider kernel functions and higher values giving narrower kernel functions. We chose to search γ values between 0.01 and 1 for all classifiers. Values between 0.01 and 0.1 were incremented by 0.01, and values above 0.1 were incremented by 0.1. Another parameter, the C parameter, controls the trade-off between the size of the decision function margin and classification accuracy, which can be thought of as a way to control overfitting the decision function. Low values of C favor a larger margin, high values of C favor a more complex decision function. We tested a range of C values from 0.1 to 10. Values between 0.1 and 1 were incremented by 0.1, and values above 1 were incremented by 1. Hyper-parameter selection for SVM classifiers trained on each type of data were optimized individually, meaning each classifier's parameter values were optimized for the data it was tested on. The pair of γ and C parameters that maximized the area under the ROC curve were used for testing the models. Supplementary Figure 2 shows the output of this procedure. Data used to train the models were standardized and scaled. Testing data given to the model were transformed based on the scalings calculated for the training data (see section 2.5.3 for more details on how data were used for classifier training and testing).

#### 2.5.2. Evaluating Classifiers

We used several standard metrics for assessing classifier performance (Lever et al., 2016), all of which describe different combinations and/or weightings of true positives (*TP*), true negatives (*TN*), false positives (*FP*), and false negatives (*FN*). For VTE identification, a *TP* is trial correctly classified as a VTE, a *TN* is a trial correctly classified as a non-VTE, a *FP* is a trial incorrectly classified as a VTE, and a *FN* is a trial incorrectly classified as a non-VTE. Accuracy measures the number of trials assigned to the correct class (VTE or non-VTE) out of the total number of trials, and is defined as:

(9) $$\begin{array}{l} accuracy=\frac{TP+TN}{TP+TN+FP+FN} \end{array}$$

such that accuracy equals 1 if every trial, VTE and non-VTE, is correctly classified, and 0 if no trials are correctly classified. Precision measures the number of correctly classified VTEs out of the total number of trials classified as a VTE, i.e.,:

(10) $$\begin{array}{l} precision=\frac{TP}{TP+FP} \end{array}$$

meaning precision takes a value of 1 if all of the trials classified as a VTE are in fact VTEs, even if it does not identify all VTEs in the dataset. As a complimentary metric, recall (also called the true positive rate) takes *FN* into account:

(11) $$\begin{array}{l} recall=\frac{TP}{TP+FN} \end{array}$$

and is thus a measure of how many VTEs were correctly classified out of the total number of VTEs in the dataset. For a binary classifier with equal numbers of each class, chance performance for each metric would be 0.5 on average.

We summarize performance by calculating the area under the ROC curve for each classifier. The ROC curve plots true positive rate (recall) and false negative rate for different probability thresholds, above which the sample is assigned to the positive class (i.e., classified as a VTE). The false negative rate is defined as:

(12) $$\begin{array}{l} FNR=\frac{FN}{TP+FN} \end{array}$$

with values of zero indicating that all VTEs were found by the classifier, and values of 1 indicating that none of the things classified as a VTE were actually VTEs. An idealized, perfect classifier will have an area under the ROC curve equal to 1, and randomly binary classification will have an area under the curve equal to 0.5 on average.

#### 2.5.3. Cross Validation

To ensure our classifiers were generalizable and performance was not biased by a particular ordering of our dataset, we performed cross-validation on randomly sampled test/train splits of the dataset. For each evaluation, we used 67% of data for supervised training, and used the remainder for testing performance. For reproducibility, and to make comparisons across classifier models and feature modalities, we created a (seeded) matrix of randomly shuffled trials where each column contained a distinct ordering of trial values to use for one split of model training and testing (Liu, X.-Y. et al., 2009). For a given assessment, we used 100 distinct splits of testing and training data, giving a matrix with 100 columns. Since VTEs occur on roughly 20% of trials, every VTE in the dataset was present in each column, and a randomly drawn, equal number of non-VTEs made up the rest of the column, meaning each distinct split used the same VTE trials, but was allowed to contain different non-VTE trials (Liu, X.-Y. et al., 2009). This same matrix was used any time we evaluated classifier performance, meaning all evaluations were done using the exact same 100 iterations of test/train splits. Put another way, we assessed performance with 100 iterations of randomly selected trials constituting each test/train split, but ensured that assessments for different classifier models and feature modalities were performed on the exact same data. The figures in this paper were generated using seed = 1.

### 2.6. Dataset Curation

Training and assessing performance of the supervised classifier required manual VTE scoring to assign labels to trials. Because it is difficult to define an exact set of criteria for scoring a VTE (hence the need for a classifier), we instead chose to have four trained raters score each trial, and used their consensus to determine the label. All raters were told to score a trajectory as a VTE if there was an indication that the rat looked toward the reward arm it did not end up choosing at least once during its trajectory. Trials where two raters scored the trajectory a VTE and two scored the trajectory a non-VTE were excluded from analysis. As shown in Supplementary Figure 1, all sessions in this dataset have an average inter-rater percent agreement above 90% and average pairwise Cohen's kappa scores above 0.7 (Hallgren, 2012; Gisev et al., 2013).

We also excluded trials based on several criteria of the hippocampal oscillation. First, we checked that the overall central tendency of the *AI* distribution was positive for a given session. Note that other studies have reported generally negative *AIs* (Amemiya and Redish, 2018; Schmidt et al., 2019). We suspect this is due to systematic shifts in theta shape characteristics across the different hippocampal axes (Buzsáki et al., 1985, 1986; Buzsáki, 2002). We also excluded trials where a 4 SD noise threshold, calculated based on the SD of the entire timeseries, was exceeded. If any session had more than 20% of its trials excluded, we did not use any of the data from that session.

### 2.7. Statistics

We performed two-sample, two-tailed Kolmogorov-Smirnov (K-S) tests to evaluate whether empirical distributions are likely drawn from the same underlying population distribution. To test whether a distribution of differences is centered at zero (i.e., to test for differences between paired groups), we performed one-sample, two-tailed Wilcoxon signed-rank tests. To assess which features exhibit statistically distinct empirical distributions when testing a number of features, we follow K-S testing with Benjamini-Hochberg (BH) false discovery rate correction to adjust *p*-values. Criteria for significance is set at *p* = 0.01 (1 divided by the number of iterations) for comparing distributions, and 0.05 for corrections. We also used Cohen's *d* metric to assess effect size, and note the suggestions that a value of 0.2 is considered a small effect, a value of 0.5 is considered a medium effect, and values above 0.8 are considered large effects (Sullivan and Feinn, 2012; Calin-Jageman, 2018). Effect sizes are denoted in text by *d*.

## 3. Results

### 3.1. Trajectory-Based Classification

**Figure 1:**
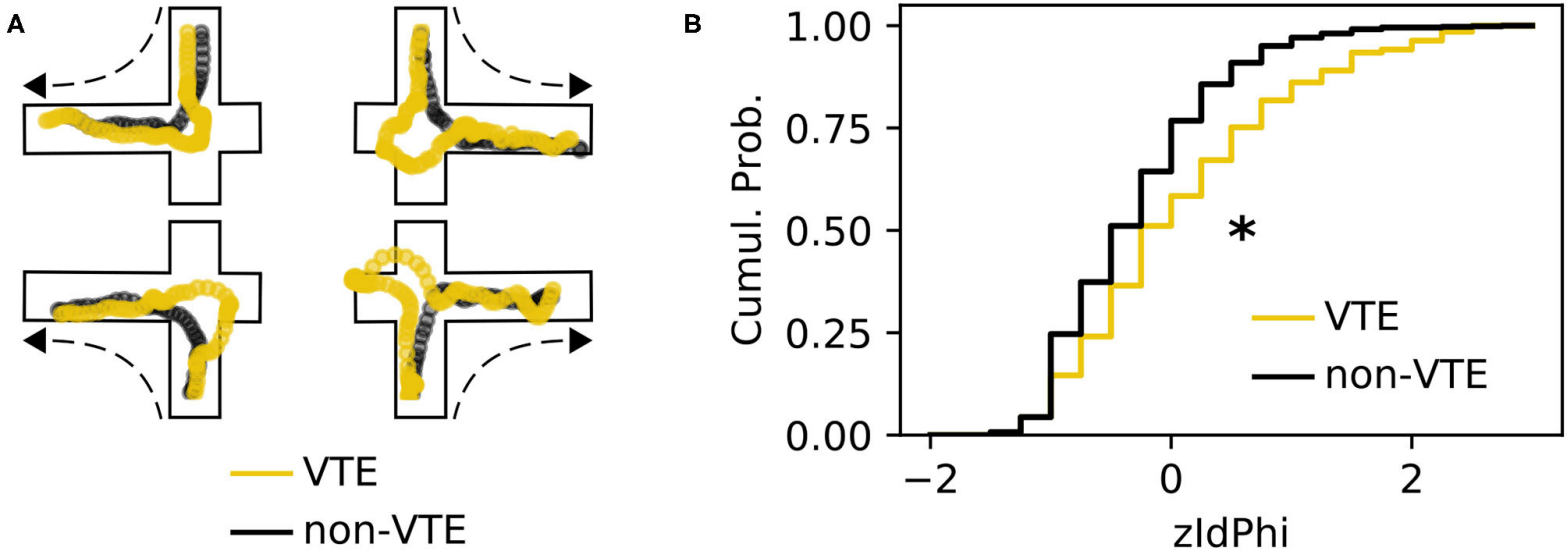
Example VTEs and zIdPhi distribution. **(A)** Example trajectories showing VTEs (yellow) and non-VTEs (black). Trajectories are shown on top of outlines of the decision-point, coming from each possible direction (shown by dashed arrow). **(B)** Empirical cumulative distributions of zIdPhi scores for VTEs (yellow) and non-VTEs (black). Note the prominent rightward shift for VTEs (*p* < 0.001, two-sample K-S test, *d* = 0.65). Cumul. Prob., cumulative probability; K-S, Kolmogorov-Smirnov.

**Figure 2:**
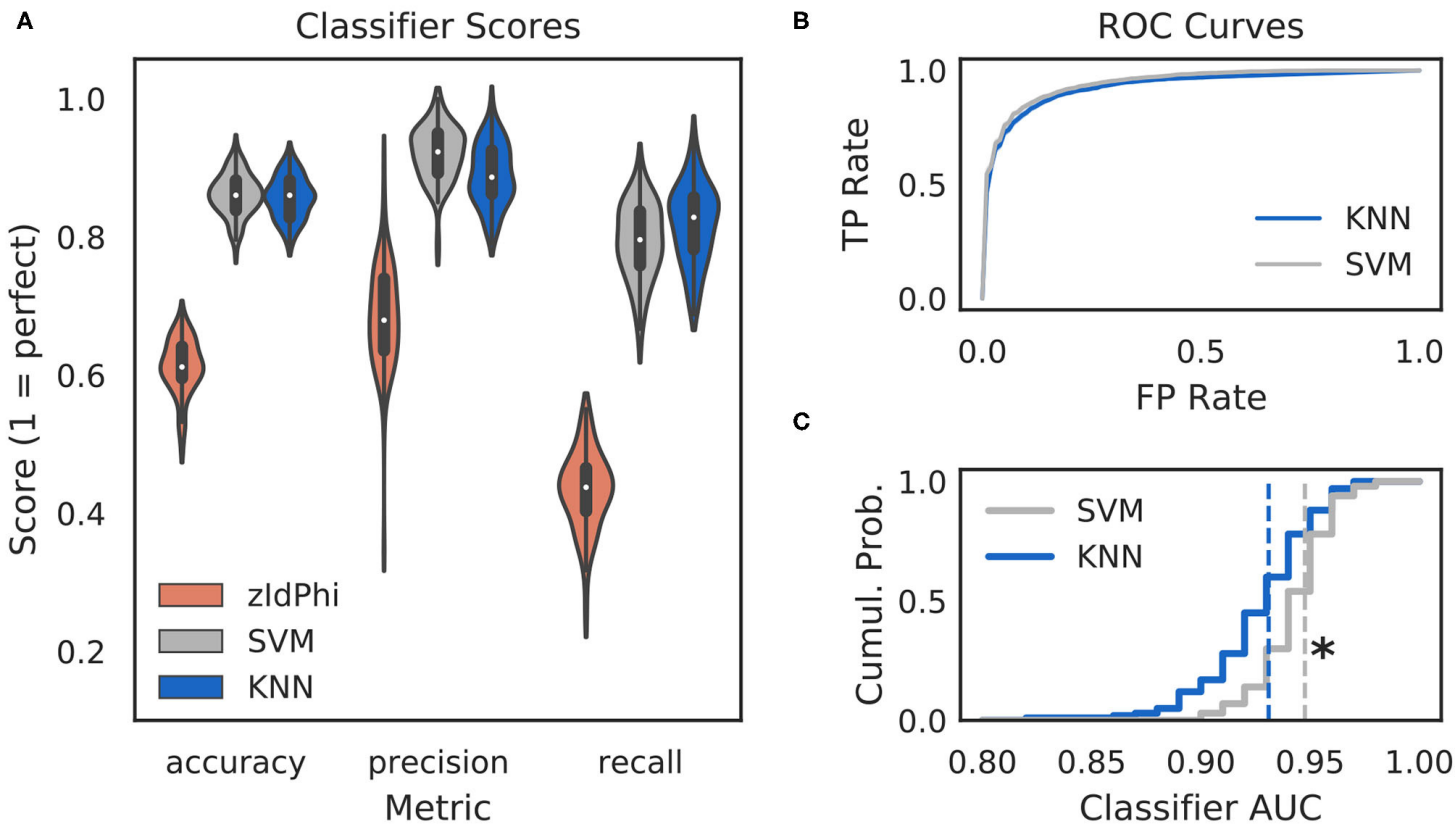
Multi-feature classifiers outperform a single metric. **(A)** Classification performance on 100 random splits of manually scored data using zIdPhi (orange), an SVM model (gray), and a KNN model (blue). Colored patches are kernel density estimates of the underlying distributions, with boxplots representing the same data inside the patches. **(B)** Receiver operating characteristic curves for KNN (blue) and SVM (gray) classifiers. Averages across splits are bold lines, 95% confidence intervals surround means, but are not visible. **(C)** Cumulative distributions of *AUC* scores for SVM in gray and KNN in blue. Note the rightward shift for the SVM model (*p* < 0.0001, two-sample K-S test, *d* = 0.78). SVM, support vector machine; KNN, k-nearest neighbor; AUC, area under the curve; TP, true positive; FP, false positive; Cumul. Prob., cumulative probability.

A VTE occurs when rats vacillate between options before their final choice. Behaviorally, this manifests as a trajectory with curves or sharp angles at choice points, where reorientations occur (Figure 1A). We analyzed a dataset with 828 trajectories from rats running a SDA task (Baker et al., 2019; Kidder et al., 2021). Each trajectory was scored as VTE (*n* = 142) or non-VTE (*n* = 686) by four trained annotators. We calculated zIdPhi (Papale et al., 2012), the z-scored, integrated change in heading angle, for each trajectory, as well as several other features (see 2.4.1 for more details). As expected, we saw statistically distinct empirical distributions for zIdPhi values on trials scored as VTE compared to non-VTE (Figure 1B, *p* < 0.001, two-sample K-S test; *d* = 0.68). When compared to manual scoring, however, using zIdPhi did not reliably separate VTE and non-VTE trials (Figure 2A).

We reasoned that we could obtain more accurate and reliable VTE detection by assessing multiple aspects of the trajectory instead of just one. As such, we calculated seven trajectory-based features (see Classifier Implementation for details) with the expectation that these features would allow for separation of VTEs and non-VTEs in a higher dimensional space. Like zIdPhi, many of these features formed distinct empirical distributions for VTEs and non-VTEs, which suggested to us that this feature set could be used to build machine learning classifiers for algorithmic VTE detection.

Classifiers are often evaluated for their accuracy, precision, and recall scores (Malley et al., 2011; Lever et al., 2016, see Classifier Implementation for detailed descriptions). In the context of VTE identification, accuracy measures the proportion of correctly labeled trials (i.e., VTE or non-VTE), precision measures the proportion of trials labeled VTE that are actually VTEs, and recall measures the proportion of VTEs found out of the total number of VTEs present. We compared performance of two widely used machine learning models—k-nearest neighbors (KNN) and support vector machines (SVM)—to zIdPhi alone in Figure 2. To generate distributions for each of these metrics, we scored 100 permutations of randomly sampled splits of data, with mutually exclusive testing and training trajectories (see Classifier Implementation for further details). To ensure scores were not influenced by the fact that we had many more non-VTE trials than VTE trials, we equalized the number of VTE and non-VTE trials for each data split. Both KNN and SVM classifiers show scores well above what would be expected by chance for accuracy (Ā_*knn*_ = 0.86, Ā_*svm*_ = 0.86; bars above letters denote mean), precision (${\bar{P}}_{knn}$ = 0.89, ${\bar{P}}_{svm}$ = 0.92), and recall (${\bar{R}}_{knn}$ = 0.82, ${\bar{R}}_{svm}$ = 0.79) on our trajectory data. Both classifier models lead to highly leftward shifted ROC curves (Figure 2B), and comparing their distributions for area under the ROC curve shows that the performance for the SVM classifier is generally higher (${\bar{AUC}}_{knn}$ = 0.93, ${\bar{AUC}}_{svm}$ = 0.95; *p* = 0.0001, two-sample K-S test; *d* = 0.78). Overall, these results suggest that we have defined a feature set suitable for VTE classification, that both KNN and SVM models provide more accurate, sensitive, and precise VTE classification than a single metric alone, and that the SVM model has a slight performance edge over the KNN model.

### 3.2. Oscillation-Based Classification

Previous research has suggested HPC involvement in decisions where VTEs occur. Early work showed that rats with bilateral HPC lesions perform less VTEs during initial learning in a visual discrimination task than rats with their hippocampi intact (Hu and Amsel, 1995). More recent research did not find differences in VTE rates for lesioned and non-lesioned animals during visual discrimination, but showed that lesioned rats exhibit fewer VTEs during early learning when performing a spatial decision-making task. In particular, lesioned rats showed fewer VTEs before finding a new reward location after it had been moved (Bett et al., 2012). Additionally, multiple studies have shown that HPC place cell activity is more likely to represent future locations during decisions involving a VTE than when no VTE occurs (Johnson and Redish, 2007; Papale et al., 2016). Furthermore, several features of the hippocampal local field potential oscillation appear to be different when decisions are made with, as opposed to without, VTEs (Amemiya and Redish, 2018; Schmidt et al., 2019).

**Figure 3:**
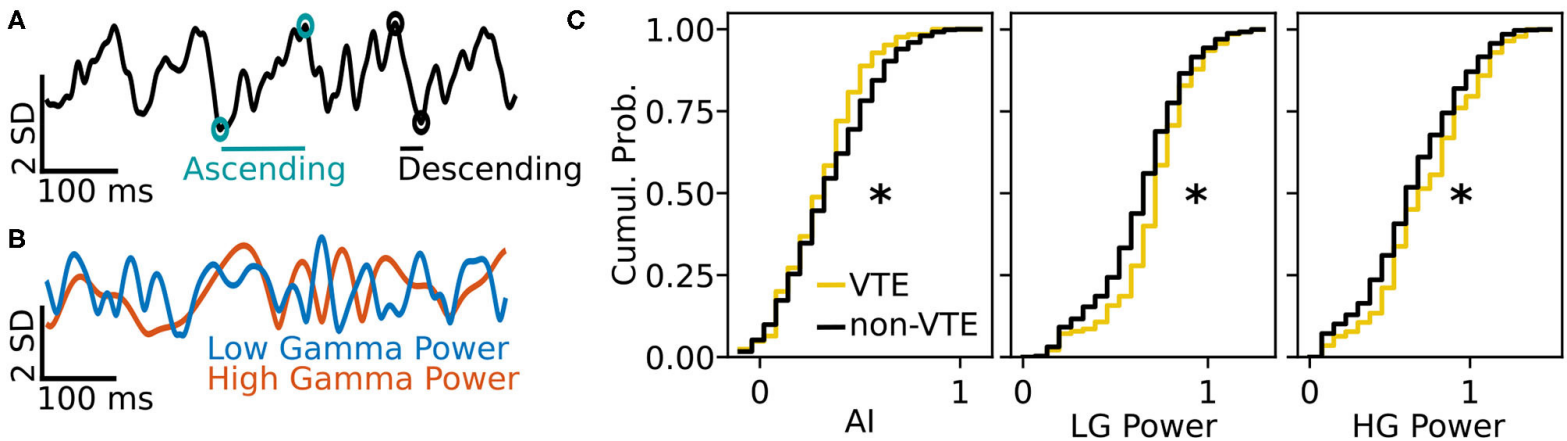
Example oscillation data and feature distributions. **(A)** Sample HPC oscillation. Data are normalized (z-scored), so amplitude is measured in standard deviations (see scale bar). Blue-green circles and line show the ascending duration of one theta cycle, and black circles and line show the descending duration of the next theta cycle. The ascending and descending durations within a single theta cycle are use to calculate the AI. **(B)** Normalized power timeseries for low gamma (blue) and high gamma (orange) for the oscillation shown in **(A)**. **(C)** Cumulative distributions for 3 (of 12) curated features of the HPC oscillation. Asterisks denote significantly different distributions between VTE and non-VTE trials (AI, *p* = 0.041; LG Power, *p* < 0.001; HG Power *p* = 0.049). AI, asymmetry index; SD, standard deviation; ms, millisecond; LG, low gamma; HG, high gamma.

**Figure 4:**
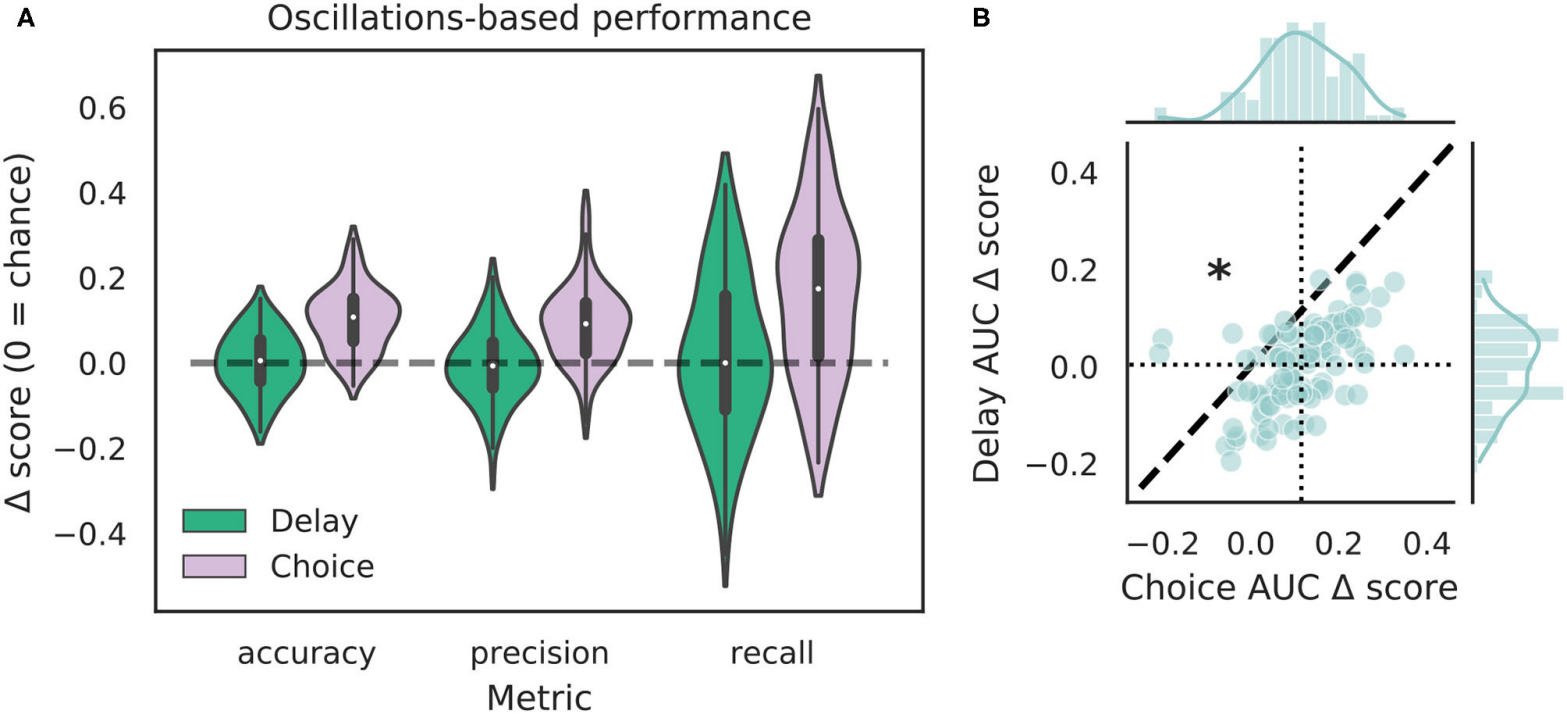
VTE classification is better for oscillation-based features from choices than delays. **(A)** Classification performance when using oscillations from the delay epoch (green) compared to oscillations from the choice epoch (purple). Each Δ score is a difference between performance using data from either epoch and corresponding randomly labeled data. **(B)** Scatterplots of Δ*AUC* score distributions for oscillations taken from the delay and choice epochs. Dotted vertical line shows the mean choice Δ*AUC* score, dotted horizontal line shows the mean delay Δ*AUC* score. Dashed diagonal line marks where equal departures from chance would occur. Individual distributions and kernel density estimates for Δ*AUC* scores are shown in the marginal distributions. Note skew below the diagonal, indicating significantly higher choice Δ*AUC* scores (*p* < 0.0001, two-sample K-S test, *d* = 1.21).

We tested how well-features of the HPC oscillation (Figure 3A) could identify VTEs using the same approach we employed for trajectory-based VTE classification. Consistent with previous work, we found several oscillatory features with different empirical distributions for VTE and non-VTE trials (Figures 3B,C). To test whether an SVM classifier could identify VTEs above chance levels when trained with features of the HPC oscillation, we calculated classifier metric Δ scores. We compared classifier performance on hippocampal data from two distinct behavioral epochs—one where rats actively made choices (i.e., when VTEs would occur), or during the delay interval that preceded the choice epoch. Each score shows how far above chance the classifier performed when oscillations were taken from the choice or delay epoch (Figure 4A, also see Supplementary Figure 3B). Chance estimates were obtained by training a classifier on oscillations from the choice epoch, but randomly labeling each trial as VTE or non-VTE. Thus, a score of zero indicates that the classifier performed the same as would be expected if randomly labeling trials. Classifier performance on the HPC oscillation during choices is above the performance for classifiers trained on the HPC oscillation during the delay epoch of the task (Figure 4B; $\Delta {\bar{AUC}}_{delay}$ = 0.004, $\Delta {\bar{AUC}}_{choice}$ = 0.11; two-sample K-S test, *p* < 0.0001; *d* = 1.21; also see Supplementary Figure 3B, pink vs. green ROC curve).

**Figure 5:**
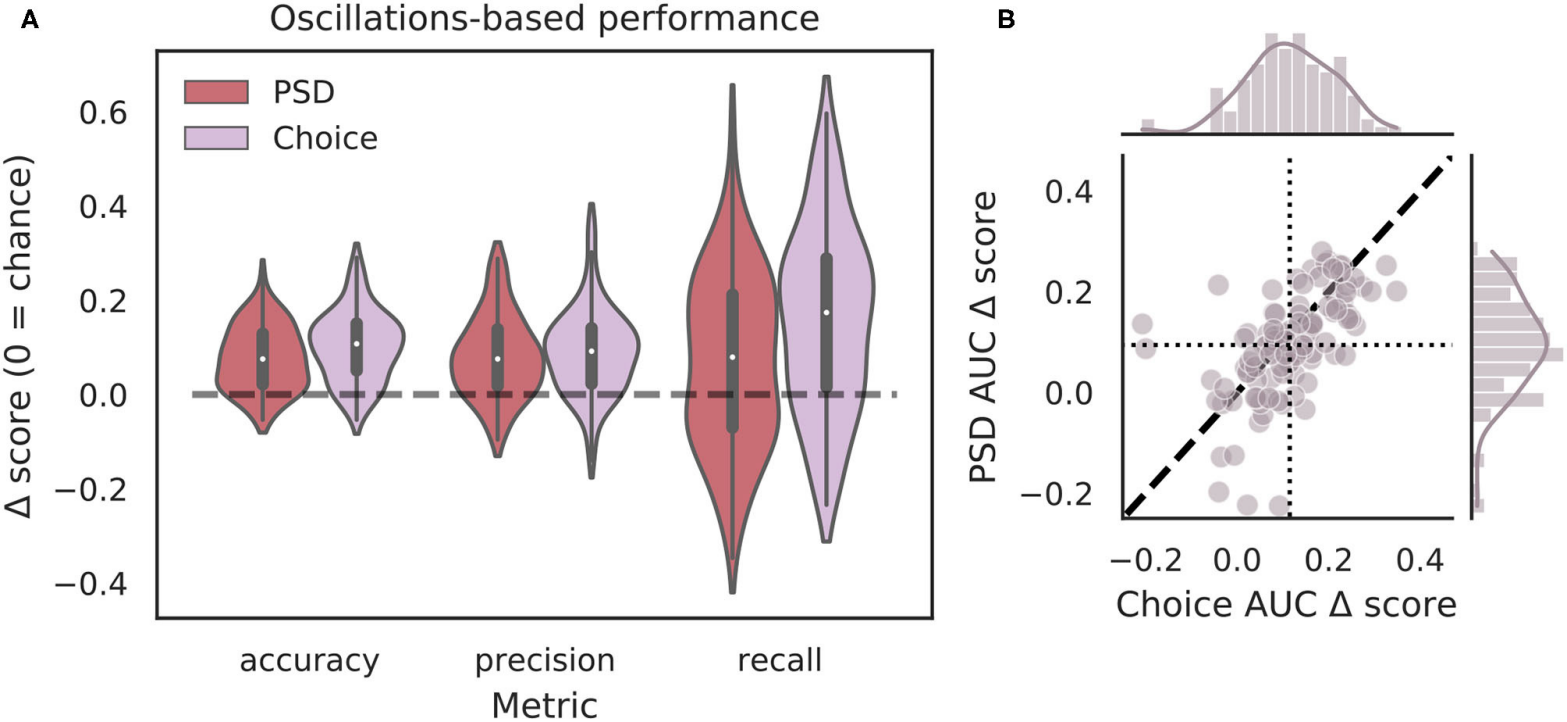
Full power spectra do not outperform highly curated oscillation features. **(A)** Classification performance when using PSD estimates (dark red) compared to curated features from the choice epoch (purple). Each Δ score is measured as a difference between performance using either PSD or oscillation-based features and corresponding randomly labeled data. **(B)** Scatterplots of Δ*AUC* score distributions for PSD estimates and oscillation-based features, both calculated for choice epochs. Dotted vertical line shows the mean choice oscillation-based Δ*AUC* score, dotted horizontal line shows the mean PSD Δ*AUC* score. Dashed diagonal line marks where equal departures from chance would occur. Individual distributions and kernel density estimates for Δ*AUC* scores are shown in the marginal plots. Δ*AUC* scores are not significantly different for SVMs trained on PSD-based and curated feature sets (*p* = 0.70, two-sample K-S test, *d* = 0.20). PSD, power spectral density.

Though the highly curated features used in the classifier for Figure 4 have been shown to differ during VTEs and non-VTEs (Amemiya and Redish, 2018; Schmidt et al., 2019), these features are only a small subset of attributes that could describe the HPC field potential oscillation. As such, we used arguably the most common descriptor of oscillations, the power spectrum, in an attempt to increase classifier performance. We first compared average power spectral density (PSD) estimates for different frequencies, calculated for different splits of data, to identify which frequencies had significantly different average power on VTE and non-VTE decisions (Supplementary Figure 4). Frequencies that survived false discovery rate correction (see section 2.7) were used as features for an SVM classifier trained on PSD estimates. Interestingly, although these classifiers utilized a much higher dimensional feature-space (roughly seven-fold more features using PSD estimates than curated oscillation-based features), Δ*AUC* scores were no different from those obtained with the highly curated features (Figure 5B, *p* = 0.70, two-sample K-S test; *d* = 0.20).

**Figure 6:**
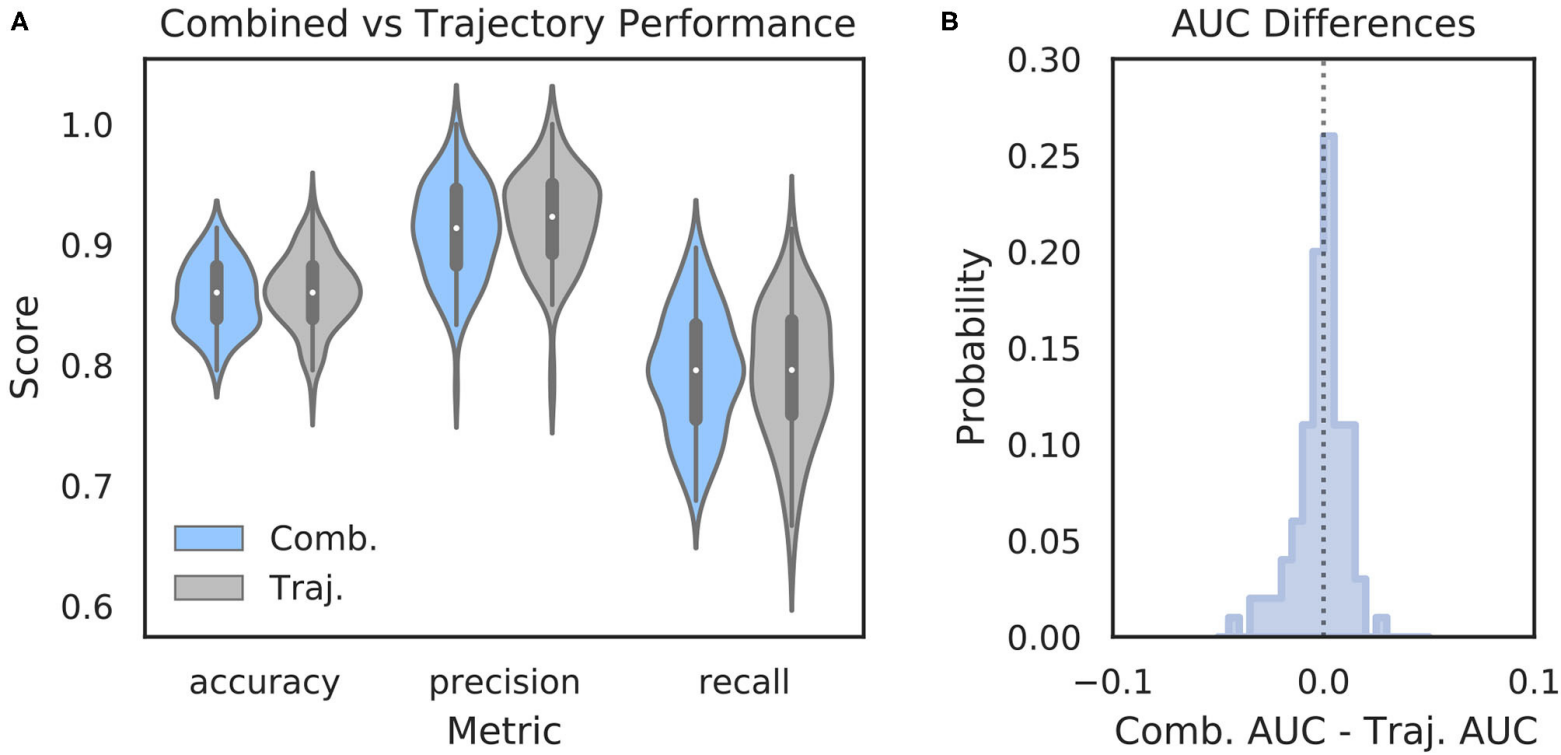
Combining oscillation- and trajectory- based features does not increase classifier performance. **(A)** Comparison of SVM classifier performance using combined oscillation- and trajectory- based features (blue) or trajectory features alone (gray, same as Figure 2A—SVM). **(B)** Distribution of *AUC* scores for classifiers trained on trajectory only features subtracted from *AUC* scores for classifiers trained on combined feature sets for corresponding data splits. Values below zero indicate worse performance using combined features. Scores are centered around zero (*p* = 0.65, Wilcoxon rank sum test, *d* = 0.08). Comb., combined; Traj., trajectory alone.

It is possible that features of the HPC oscillation contain information about VTE occurrence that complements the information contained in trajectory data. In other words, VTEs that are difficult to classify based on trajectories alone may have accompanying HPC oscillatory dynamics that, when combined with the trajectory features, lead to improved VTE classification. To examine this possibility, we trained an SVM classifier on combined trajectory- and oscillation- based features from the choice epoch that had significantly different distributions on VTE and non-VTE trials (see Supplementary Figure 4). Interestingly, combining feature sets does not change performance when compared to trajectory features alone (Figure 6; mean *AUC* difference = −0.001; *p* = 0.65, Wilcoxon signed rank test; *d* = 0.08). Thus, we conclude that, although features of the HPC oscillation can be used to some extent for classifying VTEs, these features do not contain novel or complementary information beyond what can be extracted from the trajectories.

## 4. Discussion

The purpose of this study was to improve upon current methods of VTE identification and build on our understanding of hippocampal involvement during VTEs. We show that VTE behavior can be robustly and reliably separated from non-VTE behavior using a small set of trajectory-based features. Additionally, we show that classifiers trained on features of the dorsal HPC field potential oscillation separate VTEs from non-VTEs more than would be expected by chance, supporting previous research linking the HPC to VTEs. Moreover, we show that when oscillations are taken from the delay epoch that precedes the choice epoch, oscillation-based features no longer enable above chance VTE classification, which suggests a brief temporal window underlying HPC involvement in VTE processing. We also caution, however, that despite above chance VTE identification using oscillation-based features, our results also clearly show that population level HPC dynamics are prone to VTE misclassification (Figures 4, 5), especially when compared to trajectory-based features. In particular we demonstrate that combining neural features and trajectory features does not improve performance compared to using trajectory features alone.

Not only do the small set of hippocampal oscillation features previously reported to differ between decisions where VTEs do and do not occur provide modest performance for VTE classification, using the roughly seven-fold larger feature space of the 1–100 Hz hippocampal power spectrum does not improve performance. We see this as further evidence that population level HPC dynamics only partially explain VTEs. We suspect that examining HPC interactions with other areas, such as the mPFC (Brown et al., 2016; Voss and Cohen, 2017; Schmidt et al., 2019; Hasz and Redish, 2020; Kidder et al., 2021), would be a fruitful next step for improving our ability to classify VTEs based on neural activity. Schmidt et al. have shown that rats perform fewer VTEs when faced with difficult decisions if their mPFC has been inhibited chemogenetically. Furthermore, the window in which the HPC oscillation is best able to identify VTEs is during choices, which is when brief increases in theta coherence between the dorsal HPC and mPFC occur (Jones and Wilson, 2005; Benchenane et al., 2010), suggestive of cross-regional communication (Fries, 2005, 2015). Finally, experiments using optogenetics to perturb the mPFC in a task-epoch-specific manner during the SDA task showed that stimulation decreased the proportion of VTEs rats engaged in, with a trend toward choice epoch mPFC disruption having a greater effect than stimulation in other epochs (Kidder et al., 2021).

Methodologically, we find comparing classification performance between behavior and neural activity an intuitive way to understand how well the activity under scrutiny relates to the behavior in question. The level of performance for behavior classification can often be thought of as an upper bound for assessing how well neural activity describes the behavior, while randomly labeled classifiers can set the lower bound. This may provide a more nuanced picture of how well neural activity relates to a behavior than hypothesis testing alone. For example, while we and others show multiple features of the HPC oscillation form distinct empirical distributions for VTEs and non-VTEs, the fact that classifier performance using these features does not meet classification performance of the behavior itself suggests that these features only provide a partial description about the neural substrate of the behavior. Additionally, feature-based classification allows for very flexible control of what parameters—behavioral or neural—one wishes to examine, as well as the size of the parameter space one would like to search. Moreover, as demonstrated by comparing HPC power spectra with curated oscillation features, feature vectors can be arbitrarily sized with surprisingly little influence on classifier performance, as long as the classifier is constructed to protect against overfitting (e.g., with proper hyper-parameter selection and cross-validation). For these reasons, we see this framework as extremely flexible in terms of feature selection and use, as well as an intuitive way of gauging how well neural activity measurements describe behavior.

A limitation of our study is that we do not explicitly test which features of the hippocampal oscillation are the best indicators of VTE behavior, nor do we claim that the features we test are an exhaustive list of possible features. Rather, we ask if oscillation-based features suggested by prior work can identify VTE behaviors, and to what extent they match the ability of a classifier using trajectory-based features. Similarly, this study does not address whether there is an optimal subset of power spectral density features for VTE identification. Instead, we specifically ask to what extent the range of frequencies from 1 to 100 Hz is able to identify VTE behaviors. Thus, we leave open the possibility that the hippocampal oscillation may be better able to explain VTE behaviors than is reported in this study, while suggesting a framework that others can build on to test their own hypotheses.

Altogether, our results expand previous efforts to algorithmically identify VTEs using choice trajectories from a given behavioral task, improving our ability to detect these important variants of decision-making behavior. In addition, we provide further evidence for hypotheses that situate the hippocampus as one element in what is likely a broader network of interacting neural structures supporting VTEs. We believe future decision-making research will benefit from tracking VTEs and VTE-like behaviors, such as saccades and head movements in humans and non-human primates (Voss and Cohen, 2017; Santos-Pata and Verschure, 2018) and hope our classification scheme enables more wide-spread VTE analysis. Additionally, we encourage future VTE research to expand beyond the HPC and further our understanding of the neural system(s) involved in this decision-making behavior.

## Data Availability Statement

The original contributions presented in the study are publicly available. This data can be found here: https://drive.google.com/drive/folders/1W2GFscjfDd7gFHaQrHNPNtobElY03iK8?usp=sharing.

## Ethics Statement

The animal study was reviewed and approved by University of Washington Institute for Animal Care and Use Committee.

## Author Contributions

JM, KK, DG, and SM contributed to conception and design of the study. JM, KK, ZW, and YZ annotated the data. JM analyzed the data and wrote the manuscript. All authors contributed to manuscript revision, read, and approved the submitted version.

## Conflict of Interest

The authors declare that the research was conducted in the absence of any commercial or financial relationships that could be construed as a potential conflict of interest.
