# Supplementary material for: A Machine Learning Approach for Detecting Vicarious Trial and Error Behaviors
Source: Front Neurosci. 2021 Jul 7;15:676779. doi: 10.3389/fnins.2021.676779 (PMC8292638; doi:10.3389/fnins.2021.676779)
Supplement: Supplementary file 1 [file Data_Sheet_1.PDF]

Supplemental Figures

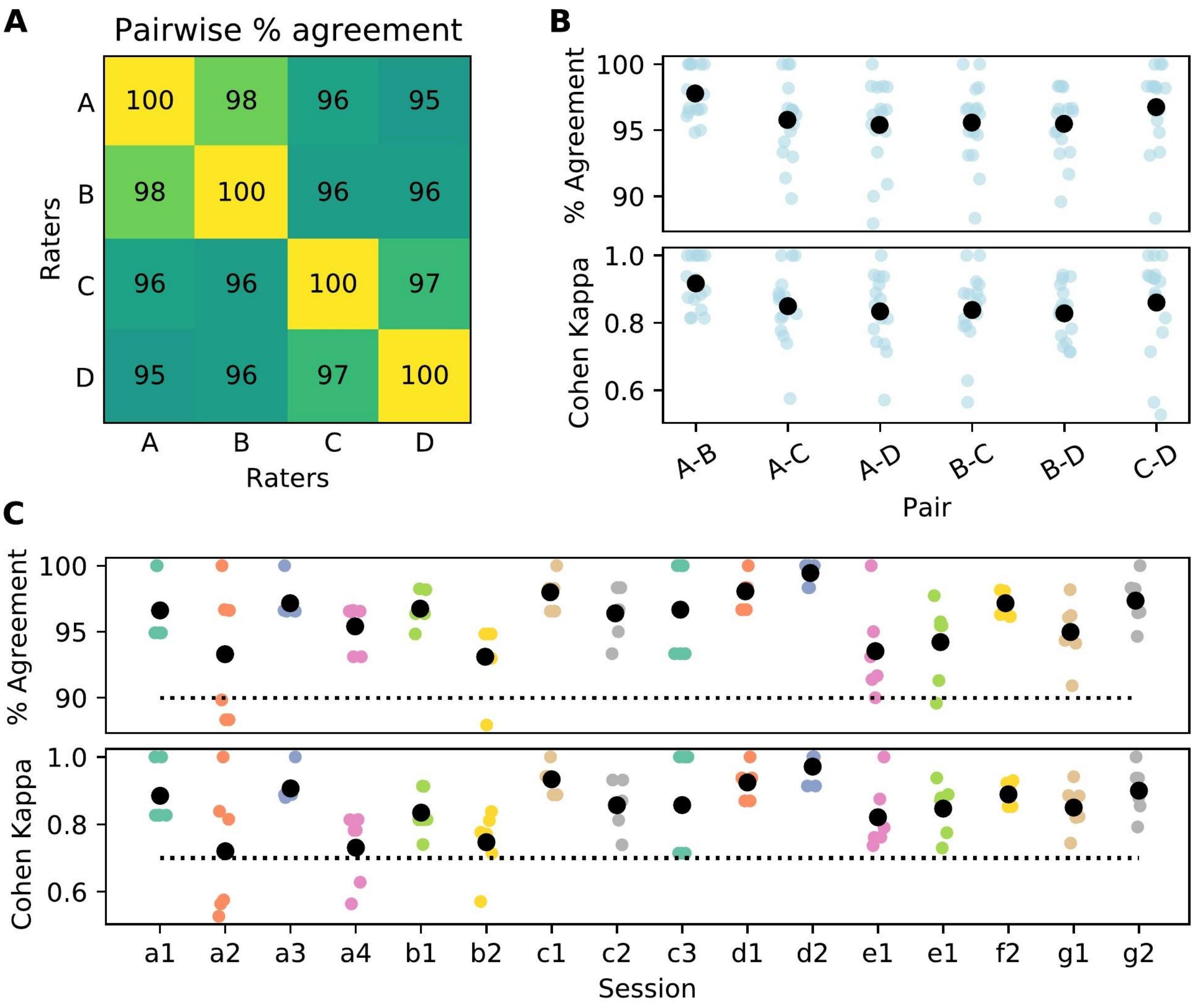

Supplemental figure 1 - *Trajectory dataset has high interrater agreement and reliability.* **A** - Pairwise % agreement between raters assessed across all trials. **B** - Top panel shows pairwise % agreement between pairs of raters on the x axis, as in **A**, but with distributions for individual sessions in blue for each pair and the mean for each session in black. Bottom panel shows Same as the top panel, but for Cohen Kappa scores using the same data shown abovet. **C** - Top panel shows pairwise percent agreement with individual sessions on the x axis and pairs of raters plotted as colored dots. Black circles show the average across pairs of raters for a given session. Bottom panel shows the same but for Cohen kappa scores.

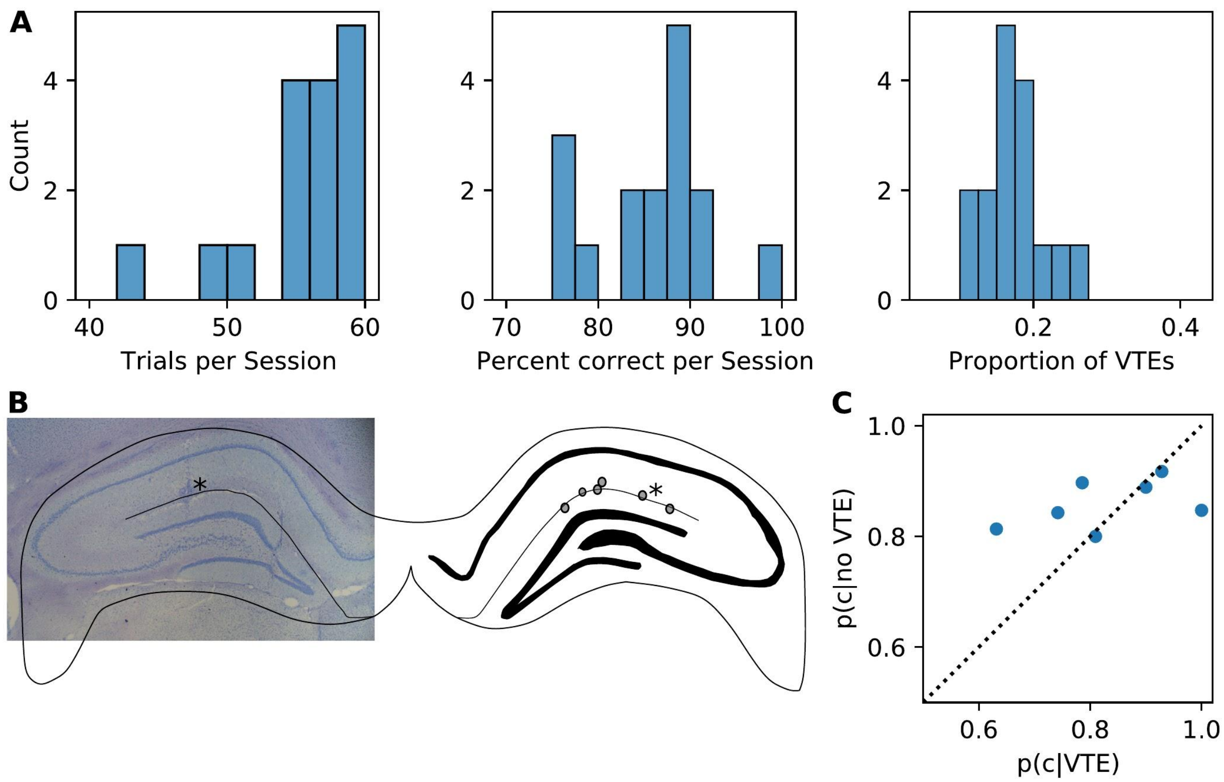

Supplemental figure 2 - *Behavioral task and tetrode location summary data.* **A** - The left panel shows a histogram of the number of trials used for classification per session, the middle panel shows a histogram of the percent of correct choices per session, and the right panel shows a histogram of the proportion of VTEs per session. **B** - The left shows an example tetrode burn mark in the hippocampal fissure, marked with an asterisk, and the right shows a schematic representation of tetrode locations from 6 of the 7 rats used in the study. The asterisk on the right corresponds to the image shown on the left. Of the 7 animals included, 3 were implanted with tetrodes in their right hemisphere, four in their left. The tetrodes were located between -3.0 and -3.6 mm posterior to bregma. **C** - Scatterplot showing the probability a rat chose correctly given that it exhibited a VTE during it's choice on the y axis against the probability it chose correctly given it did not exhibit a VTE during its choice on the x axis for the 7 rats used in this study. The dotted line indicates equal probability of choosing correctly regardless of whether a VTE occurred.

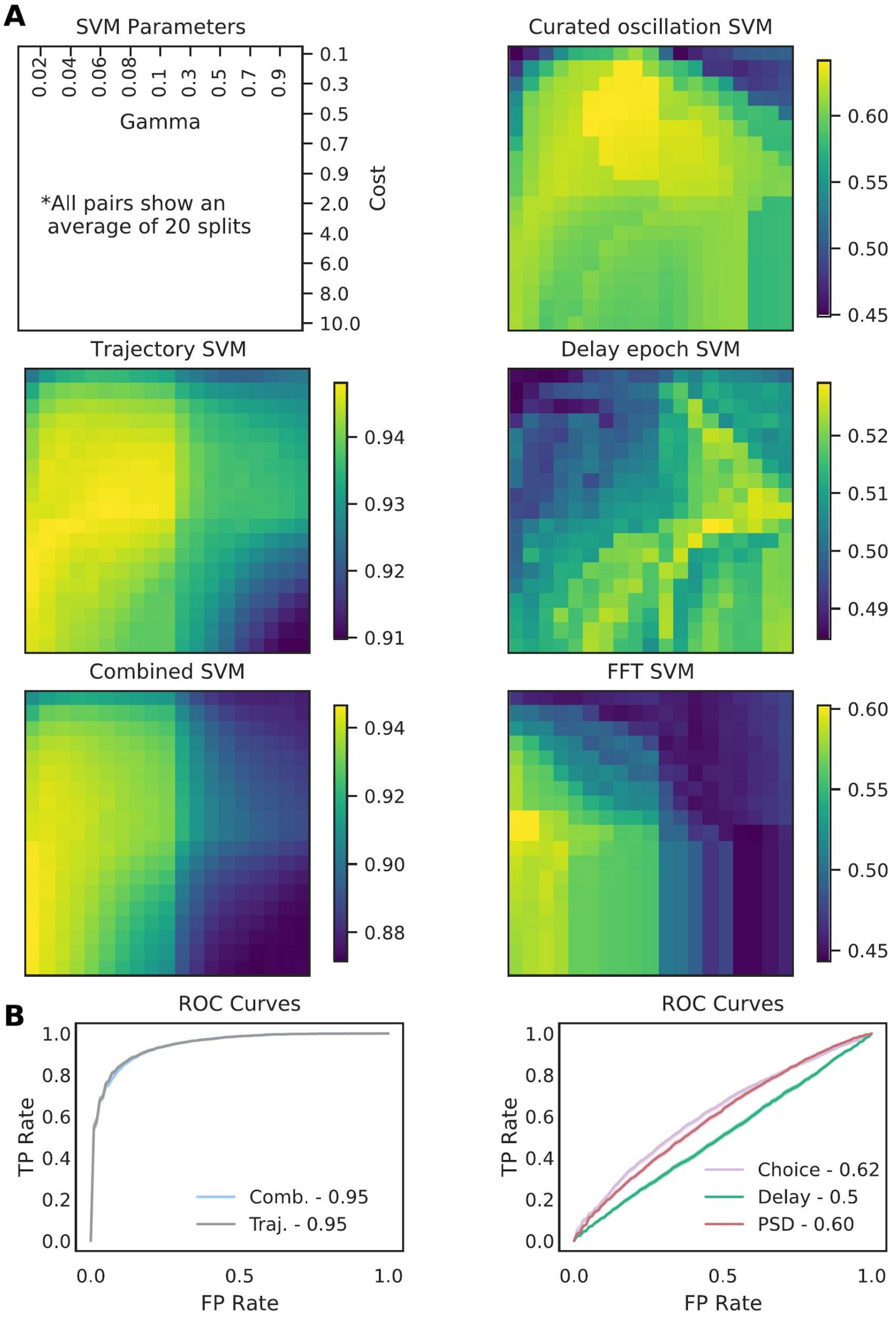
A
SVM Parameters
0.02
0.04
0.06
0.08
0.1
0.3
0.5
0.7
0.9
Gamma
0.1
0.3
0.5
0.7
0.9
2.0
4.0
6.0
8.0
10.0
Cost
*All pairs show an average of 20 splits
Curated oscillation SVM
0.60
0.55
0.50
0.45
Trajectory SVM
0.94
0.93
0.92
0.91
Delay epoch SVM
0.52
0.51
0.50
0.49
Combined SVM
0.94
0.92
0.90
0.88
FFT SVM
0.60
0.55
0.50
0.45
B
ROC Curves
TP Rate
FP Rate
Comb. - 0.95
Traj. - 0.95
ROC Curves
TP Rate
FP Rate
Choice - 0.62
Delay - 0.5
PSD - 0.60

Supplemental figure 3 - *SVM classifier hyperparameter optimization and ROC curves.* **A** - Heatmaps showing area under the ROC curve for SVM classifiers trained on different features with different parameter pairs. The parameter values are diagrammed in the top left panel of the figure. Each colored square represents the mean of 20 different splits of data, with warmer colors representing greater area under the curve. Colorbars for each heatmap are to the right of the panel. Heatmaps on the right side show area under the ROC curve for oscillation-based features, heatmaps on the left show area under the ROC curve trajectory (middle) or combined trajectory- and oscillation-based features. **B** - Full ROC curves for the different classifiers using the parameter pair with the maximum value from **A**. The left compares classification with trajectory only (grey) or combined trajectory- and classification- based features. Numbers next to the legend labels are the average area under the curve for each feature set.

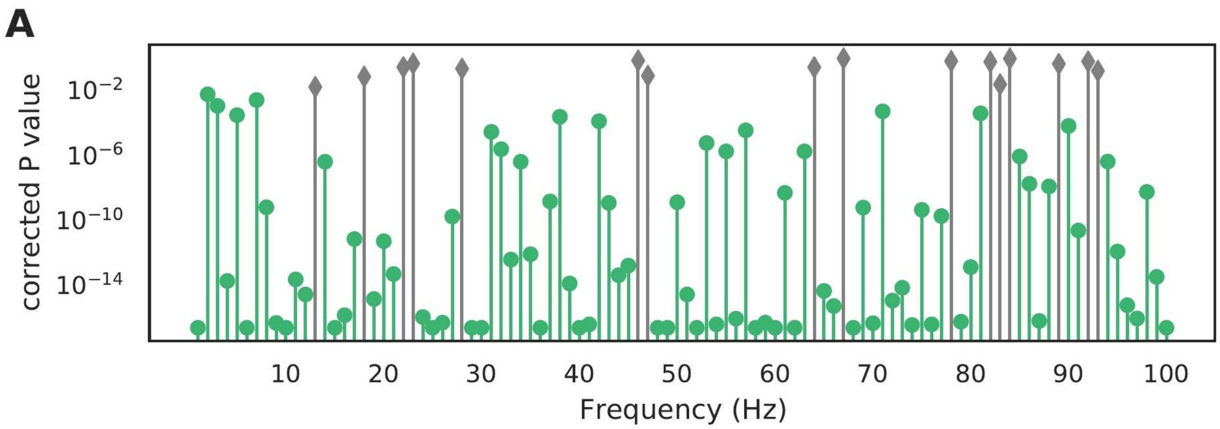

Supplemental figure 4 - *Power spectral density frequencies that survive false discovery rate correction*. **A** - Stem plot of power spectral densities (PSD) estimated to be significantly different on VTE and non-VTE trials. PSDs are estimated for 100 different trial permutations, each containing the same number of VTEs and non-VTEs, and the mean of that permutation across frequencies makes one member of a distribution used for hypothesis testing. A p-value is calculated for each frequency's distribution using a Wilcoxon signed rank test to see if the difference between distributions for VTE and non-VTE trials is centered at 0. Then, each frequency distribution's p-value is corrected using the Benjamini-Hochberg false discovery rate correction for multiple comparisons based on an alpha value of 0.05. Green stems with circles show frequencies that are considered statistically significant after correction, grey stems with diamonds show frequencies that are not considered statistically significant after correction.
